# *trans*-Bis[1-(2-benzamido­eth­yl)-3-(2,4,6-trimethyl­phen­yl)imidazol-2-yl­idene]dichloridopalladium(II)

**DOI:** 10.1107/S1600536812031868

**Published:** 2012-07-18

**Authors:** Stefan Warsink, Andreas Roodt

**Affiliations:** a Department of Chemistry, University of the Free State, PO Box 339, Bloemfontein, South Africa

## Abstract

In the title compound, [PdCl_2_(C_21_H_23_N_3_O)_2_], the Pd^II^ atom is located on an inversion centre and is coordinated in a slightly distorted square-planar environment by the chloride and *N*-heterocyclic carbene (NHC) ligands in mutual *trans* positions. There are several hydrogen-bonding inter­actions, the most significant of which is a hydrogen bond between the amide moiety of the NHC and the chloride ligand. These hydrogen-bond interactions form a three-dimensional network.

## Related literature

For a review on *N*-heterocyclic carbenes (NHCs) and their coordination chemistry, see: Hahn & Jahnke (2008 ▶). For seminal papers on NHC structure and coordination chemistry, see: Arduengo *et al.* (1991 ▶); Wang & Lin (1998 ▶). For Pd(NHC) complexes, see, for example: Meij *et al.* (2005 ▶); Warsink *et al.* (2009 ▶, 2010 ▶); Fu *et al.* (2010 ▶).
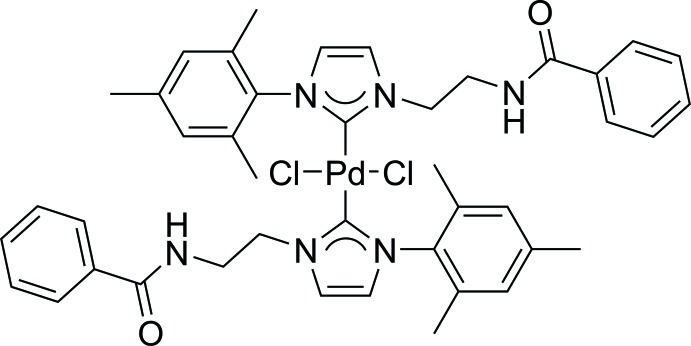


## Experimental

### 

#### Crystal data

[PdCl_2_(C_21_H_23_N_3_O)_2_]
*M* *_r_* = 844.15
Monoclinic, 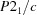

*a* = 12.594 (4) Å
*b* = 11.736 (4) Å
*c* = 14.403 (4) Å
β = 113.3098 (10)°
*V* = 1955.0 (10) Å^3^
*Z* = 2
Mo *K*α radiation
μ = 0.66 mm^−1^
*T* = 100 K
0.73 × 0.58 × 0.25 mm

#### Data collection

Bruker X8 APEXII KappaCCD diffractometer
Absorption correction: multi-scan (*SADABS*; Bruker, 2007 ▶) *T*_min_ = 0.640, *T*_max_ = 0.849
28432 measured reflections
4845 independent reflections
4441 reflections with *I* > 2σ(*I*)
*R*_int_ = 0.025

#### Refinement

*R*[*F* ^2^ > 2σ(*F* ^2^)] = 0.021
*wR*(*F* ^2^) = 0.055
*S* = 1.03
4845 reflections
248 parameters
H atoms treated by a mixture of independent and constrained refinement
Δρ_max_ = 0.37 e Å^−3^
Δρ_min_ = −0.43 e Å^−3^

### 

Data collection: *APEX2* (Bruker, 2007 ▶); cell refinement: *SAINT-Plus* (Bruker, 2007 ▶); data reduction: *SAINT-Plus*; program(s) used to solve structure: *SIR97* (Altomare *et al.*, 1999 ▶); program(s) used to refine structure: *SHELXL97* (Sheldrick, 2008 ▶); molecular graphics: *Mercury* (Macrae *et al.*, 2008 ▶); software used to prepare material for publication: *WinGX* (Farrugia, 1999 ▶).

## Supplementary Material

Crystal structure: contains datablock(s) I, global. DOI: 10.1107/S1600536812031868/wm2660sup1.cif

Structure factors: contains datablock(s) I. DOI: 10.1107/S1600536812031868/wm2660Isup2.hkl

Additional supplementary materials:  crystallographic information; 3D view; checkCIF report

## Figures and Tables

**Table:** 

C01—Pd1	2.0335 (14)
Cl1—Pd1	2.3188 (6)

**Table:** 

C01—Pd1—Cl1	87.55 (4)

**Table 2:** Hydrogen-bond geometry (Å, °)

*D*—H⋯*A*	*D*—H	H⋯*A*	*D*⋯*A*	*D*—H⋯*A*
N3—H1⋯Cl1^i^	0.797 (19)	2.549 (19)	3.3181 (15)	162.6 (17)
C09—H09⋯O1^ii^	0.95	2.49	3.386 (2)	157
C10—H10⋯O1^iii^	0.95	2.44	3.201 (2)	137
C19—H19*C*⋯Cl1	0.98	2.81	3.772 (2)	167
C21—H21*B*⋯Cl1^i^	0.98	2.78	3.567 (2)	137

Symmetry codes: (i) 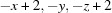; (ii) 
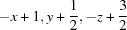
; (iii) 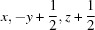.

**Fig. 1.**
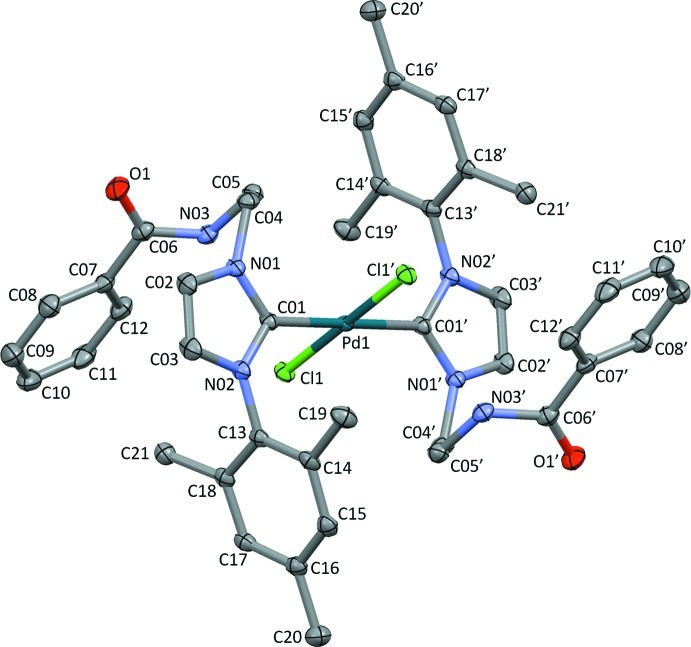
The molecular structure of compound (I) with displacement ellipsoids drawn at the 50% probability level. Hydrogen atoms have been omitted for clarity. [Symmetry code to generate symmetry-related atoms marked with ' : -x + 2, -y, -z + 2.]

## supplementary crystallographic information

### Comment

Palladium complexes bearing NHC ligands are well documented in literature (Hahn & Jahnke, 2008), even before the first free NHC was crystallographically characterized (Arduengo *et al.*, 1991). As part of our focus on ligand manipulation for palladium complexes (Meij *et al.*, 2005), we concentrated on the development of complexes bearing NHC ligands. NHCs are well-known for being very good σ-donors, but because of the empty *p*-orbital on the carbene carbon, complexes with high electron density are not necessarily destabilized by the presence of an NHC (Warsink *et al.*, 2010). Several examples exist where an NHC is present on an electron-rich palladium(0) atom (Warsink *et al.*, 2009), or where more than one NHC is present on palladium(II) (Fu *et al.*, 2010).

With the addition of two NHCs to palladium(II), two possible isomers can result. Both have been prepared, with reaction conditions normally favouring the kinetic *trans*-product. The *cis*-product can be obtained by performing the reaction under thermodynamic control. When this type of complex is prepared from the silver(I) NHC complex (Wang & Lin, 1998), transfer of the carbene ligand usually takes place in minutes, even when two NHC moieties are transferred. The precipitation of the silver salt ensures the reaction goes to completion.

The geometric parameters of the title compound, [PdCl_2_(C_21_H_23_N_3_O)_2_], (I), show that the complex is square-planar, with bond lengths between palladium and its ligands being in the expected range. The Pd^2+^ cation lies on an inversion centre, generating half of the molecule by symmetry. The C1—Pd1—Cl1 angle is 87.55 (4) °, slightly distorting the geometry of the complex. The NHC is twisted out of the coordination plane to alleviate the steric bulk induced by the mesityl-substituent; the dihedral angle between the carbene core and the coordination plane is 72.37 (13) °.

There are several hydrogen bonding interactions, both inter- and intramolecular. The most significant of these is a hydrogen bond between the amide H atoms and the chlorido ligands (Table 2)

### Experimental

To a dichloromethane solution of chlorido[(1-(2-benzamido)-ethylene-3-mesityl)-imidazol-2-ylidene]silver(I) (0.175 g, 0.36 mmol) was added 0.5 equivalent of dichlorido bis(acetonitrile)palladium(II). The resulting orange solution changed to a suspension in 5 minutes time. This suspension was filtered over a celite pad and the pale yellow solution was concentrated to give the product as a pale orange solid in a yield of 98% (150 mg). ^1^H NMR (300 MHz, CDCl_3_): δ 7.75 (d, ^3^*J*(HH) = 7.4 Hz, 4H, *o*-Ph—H), 7.45 (t, ^3^*J*(HH) = 7.3 Hz, 2H, *p*-Ph—H), 7.35 (dt, ^3^*J*(HH) = 7.4 Hz, ^3^*J*(HH) = 7.3 Hz, 4H, *m*-Ph—H), 7.08 (broad t, ^3^*J*(HH) = 5.9 Hz, 2H, NH), 6.93 (s, 4H, Mes-H), 6.89 (d, ^3^*J*(HH) = 1.6 Hz, 2H, CH), 6.69 (d, ^3^*J*(HH) = 1.6 Hz, 2H, CH), 4.41 (t, ^3^*J*(HH) = 5.6 Hz, 4H, NCH_2_), 4.02 (dt, ^3^*J*(HH) = 5.6 Hz, ^3^*J*(HH) = 5.9 Hz, 4H, NHCH_2_), 2.31 (s, 6H, *p*-Mes-CH_3_), 2.09 (s, 12H, *o*-Mes-CH_3_). Colourless crystals were obtained by vapour diffusion of diethyl ether into a concentrated dichloromethane solution.

### Crystal data

**Table:**

[PdCl_2_(C_21_H_23_N_3_O)_2_]	*F*(000) = 872
*M**_r_* = 844.15	*D*_x_ = 1.434 Mg m^−^^3^
Monoclinic, *P*2_1_/*c*	Mo *K*α radiation, λ = 0.71073 Å
Hall symbol: -P 2ybc	Cell parameters from 9963 reflections
*a* = 12.594 (4) Å	θ = 2.5–28.3°
*b* = 11.736 (4) Å	µ = 0.66 mm^−^^1^
*c* = 14.403 (4) Å	*T* = 100 K
β = 113.3098 (10)°	Plate, colourless
*V* = 1955.0 (10) Å^3^	0.73 × 0.58 × 0.25 mm
*Z* = 2	

### Data collection

**Table:**

Bruker X8 APEXII KappaCCD diffractometer	4845 independent reflections
Radiation source: sealed tube	4441 reflections with *I* > 2σ(*I*)
Graphite monochromator	*R*_int_ = 0.025
φ and ω scans	θ_max_ = 28.3°, θ_min_ = 1.8°
Absorption correction: multi-scan (*SADABS*; Bruker, 2007)	*h* = −15→16
*T*_min_ = 0.640, *T*_max_ = 0.849	*k* = −15→15
28432 measured reflections	*l* = −19→19

### Refinement

**Table:**

Refinement on *F*^2^	Primary atom site location: structure-invariant direct methods
Least-squares matrix: full	Secondary atom site location: difference Fourier map
*R*[*F*^2^ > 2σ(*F*^2^)] = 0.021	Hydrogen site location: inferred from neighbouring sites
*wR*(*F*^2^) = 0.055	H atoms treated by a mixture of independent and constrained refinement
*S* = 1.03	*w* = 1/[σ^2^(*F*_o_^2^) + (0.0252*P*)^2^ + 1.206*P*] where *P* = (*F*_o_^2^ + 2*F*_c_^2^)/3
4845 reflections	(Δ/σ)_max_ = 0.001
248 parameters	Δρ_max_ = 0.37 e Å^−^^3^
0 restraints	Δρ_min_ = −0.43 e Å^−^^3^
0 constraints	

### Special details

**Table:**

Experimental. The intensity data was collected on a Bruker X8 ApexII 4 K Kappa CCD diffractometer using an exposure time of 5 s/frame. A total of 1386 frames was collected with a frame width of 0.5° covering up to θ = 28.31° with 99.7% completeness accomplished.
Geometry. All s.u.'s (except the s.u. in the dihedral angle between two l.s. planes) are estimated using the full covariance matrix. The cell s.u.'s are taken into account individually in the estimation of s.u.'s in distances, angles and torsion angles; correlations between s.u.'s in cell parameters are only used when they are defined by crystal symmetry. An approximate (isotropic) treatment of cell s.u.'s is used for estimating s.u.'s involving l.s. planes.
Refinement. Refinement of *F*^2^ against ALL reflections. The weighted *R*-factor *wR* and goodness of fit *S* are based on *F*^2^, conventional *R*-factors *R* are based on *F*, with *F* set to zero for negative *F*^2^. The threshold expression of *F*^2^ > 2σ(*F*^2^) is used only for calculating *R*-factors(gt) *etc*. and is not relevant to the choice of reflections for refinement. *R*-factors based on *F*^2^ are statistically about twice as large as those based on *F*, and *R*- factors based on ALL data will be even larger.

### Fractional atomic coordinates and isotropic or equivalent isotropic displacement parameters (Å^2^)

**Table:**

	*x*	*y*	*z*	*U*_iso_*/*U*_eq_	
C01	0.93466 (11)	0.12682 (11)	0.89676 (9)	0.0151 (2)	
C02	0.83145 (13)	0.21976 (12)	0.75160 (10)	0.0225 (3)	
H02	0.7778	0.2338	0.6844	0.027*	
C03	0.90016 (13)	0.29692 (13)	0.81658 (10)	0.0237 (3)	
H03	0.905	0.3758	0.8041	0.028*	
C04	0.79774 (13)	0.00921 (12)	0.75366 (10)	0.0195 (3)	
H04A	0.855	−0.0393	0.7411	0.023*	
H04B	0.7351	0.0269	0.6874	0.023*	
C05	0.74689 (12)	−0.05763 (12)	0.81778 (10)	0.0204 (3)	
H05A	0.687	−0.1103	0.7731	0.025*	
H05B	0.8089	−0.1045	0.8673	0.025*	
C06	0.60980 (12)	0.08742 (13)	0.82388 (10)	0.0207 (3)	
C07	0.57994 (12)	0.16735 (13)	0.89085 (10)	0.0204 (3)	
C08	0.54646 (13)	0.27752 (14)	0.85645 (11)	0.0269 (3)	
H08	0.5419	0.2997	0.7915	0.032*	
C09	0.51965 (15)	0.35543 (15)	0.91642 (13)	0.0336 (4)	
H09	0.4984	0.4312	0.8932	0.04*	
C10	0.52404 (14)	0.32240 (17)	1.01020 (12)	0.0345 (4)	
H10	0.5057	0.3756	1.0513	0.041*	
C11	0.55517 (13)	0.21202 (16)	1.04414 (11)	0.0305 (4)	
H11	0.5565	0.1894	1.1079	0.037*	
C12	0.58435 (12)	0.13441 (14)	0.98548 (10)	0.0240 (3)	
H12	0.6072	0.0592	1.0096	0.029*	
C13	1.04514 (12)	0.29318 (11)	0.99505 (9)	0.0169 (2)	
C14	1.16210 (12)	0.29244 (11)	1.01144 (10)	0.0189 (3)	
C15	1.23975 (12)	0.34349 (12)	1.09968 (11)	0.0205 (3)	
H15	1.3197	0.3448	1.1123	0.025*	
C16	1.20260 (12)	0.39272 (12)	1.16990 (10)	0.0201 (3)	
C17	1.08462 (12)	0.39442 (11)	1.14869 (10)	0.0191 (3)	
H17	1.0589	0.4282	1.1961	0.023*	
C18	1.00316 (12)	0.34792 (11)	1.05991 (10)	0.0175 (3)	
C19	1.20317 (13)	0.23864 (13)	0.93643 (11)	0.0254 (3)	
H19A	1.2861	0.253	0.9574	0.038*	
H19B	1.1608	0.2717	0.8693	0.038*	
H19C	1.1893	0.1563	0.9339	0.038*	
C20	1.28891 (13)	0.44417 (14)	1.26630 (11)	0.0269 (3)	
H20A	1.2478	0.4878	1.2998	0.04*	
H20B	1.3412	0.4948	1.2503	0.04*	
H20C	1.3337	0.3833	1.3113	0.04*	
C21	0.87535 (12)	0.36035 (12)	1.03310 (10)	0.0212 (3)	
H21A	0.863	0.3995	1.0881	0.032*	
H21B	0.8395	0.2847	1.023	0.032*	
H21C	0.8404	0.4049	0.9708	0.032*	
N1	0.85384 (10)	0.11576 (10)	0.80130 (8)	0.0165 (2)	
N2	0.96271 (10)	0.23883 (9)	0.90550 (8)	0.0167 (2)	
N3	0.69588 (11)	0.01305 (10)	0.87193 (9)	0.0197 (2)	
O1	0.55957 (10)	0.09075 (10)	0.73122 (7)	0.0289 (2)	
Cl1	1.09679 (3)	−0.06511 (3)	0.90286 (2)	0.01787 (7)	
Pd1	1	0	1	0.01239 (4)	
H1	0.7338 (16)	0.0244 (15)	0.9304 (15)	0.023 (4)*	

### Atomic displacement parameters (Å^2^)

**Table:**

	*U*^11^	*U*^22^	*U*^33^	*U*^12^	*U*^13^	*U*^23^
C01	0.0188 (6)	0.0141 (6)	0.0136 (5)	0.0012 (5)	0.0077 (5)	−0.0003 (4)
C02	0.0312 (7)	0.0195 (7)	0.0146 (6)	0.0054 (6)	0.0068 (5)	0.0044 (5)
C03	0.0368 (8)	0.0166 (7)	0.0163 (6)	0.0037 (6)	0.0091 (6)	0.0055 (5)
C04	0.0233 (7)	0.0188 (7)	0.0146 (6)	−0.0007 (5)	0.0056 (5)	−0.0040 (5)
C05	0.0229 (7)	0.0169 (7)	0.0188 (6)	−0.0006 (5)	0.0053 (5)	−0.0006 (5)
C06	0.0210 (6)	0.0232 (7)	0.0164 (6)	−0.0015 (5)	0.0058 (5)	0.0010 (5)
C07	0.0175 (6)	0.0260 (7)	0.0143 (6)	−0.0009 (5)	0.0029 (5)	−0.0015 (5)
C08	0.0288 (7)	0.0270 (8)	0.0209 (7)	0.0035 (6)	0.0055 (6)	0.0021 (6)
C09	0.0346 (9)	0.0270 (8)	0.0303 (8)	0.0060 (7)	0.0034 (7)	−0.0040 (6)
C10	0.0276 (8)	0.0436 (10)	0.0253 (8)	0.0059 (7)	0.0029 (6)	−0.0146 (7)
C11	0.0251 (7)	0.0485 (10)	0.0155 (6)	0.0039 (7)	0.0054 (6)	−0.0040 (6)
C12	0.0212 (7)	0.0320 (8)	0.0164 (6)	0.0010 (6)	0.0048 (5)	0.0016 (6)
C13	0.0239 (6)	0.0110 (6)	0.0149 (6)	−0.0006 (5)	0.0068 (5)	0.0015 (4)
C14	0.0256 (7)	0.0125 (6)	0.0211 (6)	−0.0006 (5)	0.0119 (5)	0.0004 (5)
C15	0.0229 (7)	0.0150 (6)	0.0240 (7)	−0.0004 (5)	0.0097 (5)	0.0008 (5)
C16	0.0261 (7)	0.0132 (6)	0.0190 (6)	−0.0010 (5)	0.0069 (5)	0.0008 (5)
C17	0.0275 (7)	0.0134 (6)	0.0176 (6)	−0.0003 (5)	0.0104 (5)	−0.0006 (5)
C18	0.0247 (7)	0.0117 (6)	0.0172 (6)	−0.0001 (5)	0.0093 (5)	0.0024 (5)
C19	0.0301 (8)	0.0236 (7)	0.0284 (7)	−0.0035 (6)	0.0179 (6)	−0.0049 (6)
C20	0.0275 (7)	0.0265 (8)	0.0233 (7)	−0.0030 (6)	0.0064 (6)	−0.0053 (6)
C21	0.0248 (7)	0.0190 (7)	0.0211 (6)	0.0023 (5)	0.0106 (5)	0.0006 (5)
N1	0.0216 (5)	0.0157 (5)	0.0122 (5)	0.0019 (4)	0.0066 (4)	0.0005 (4)
N2	0.0233 (6)	0.0131 (5)	0.0135 (5)	0.0010 (4)	0.0071 (4)	0.0018 (4)
N3	0.0208 (6)	0.0233 (6)	0.0130 (5)	0.0001 (5)	0.0045 (5)	0.0008 (4)
O1	0.0297 (6)	0.0383 (6)	0.0134 (4)	0.0082 (5)	0.0029 (4)	−0.0008 (4)
Cl1	0.02339 (15)	0.01741 (15)	0.01444 (13)	0.00286 (12)	0.00921 (11)	−0.00015 (11)
Pd1	0.01673 (7)	0.01039 (7)	0.00985 (7)	0.00060 (5)	0.00504 (5)	0.00040 (4)

### Geometric parameters (Å, º)

**Table:**

C01—N2	1.3541 (18)	C11—H11	0.95
C01—N1	1.3548 (16)	C12—H12	0.95
C01—Pd1	2.0335 (14)	C13—C14	1.397 (2)
C02—C03	1.343 (2)	C13—C18	1.3985 (19)
C02—N1	1.3864 (18)	C13—N2	1.4451 (17)
C02—H02	0.95	C14—C15	1.395 (2)
C03—N2	1.3882 (17)	C14—C19	1.5085 (19)
C03—H03	0.95	C15—C16	1.396 (2)
C04—N1	1.4663 (18)	C15—H15	0.95
C04—C05	1.532 (2)	C16—C17	1.394 (2)
C04—H04A	0.99	C16—C20	1.510 (2)
C04—H04B	0.99	C17—C18	1.3955 (19)
C05—N3	1.4512 (19)	C17—H17	0.95
C05—H05A	0.99	C18—C21	1.506 (2)
C05—H05B	0.99	C19—H19A	0.98
C06—O1	1.2307 (17)	C19—H19B	0.98
C06—N3	1.3498 (19)	C19—H19C	0.98
C06—C07	1.496 (2)	C20—H20A	0.98
C07—C08	1.389 (2)	C20—H20B	0.98
C07—C12	1.3965 (19)	C20—H20C	0.98
C08—C09	1.388 (2)	C21—H21A	0.98
C08—H08	0.95	C21—H21B	0.98
C09—C10	1.385 (3)	C21—H21C	0.98
C09—H09	0.95	N3—H1	0.797 (19)
C10—C11	1.385 (3)	Cl1—Pd1	2.3188 (6)
C10—H10	0.95	Pd1—C01^i^	2.0335 (14)
C11—C12	1.387 (2)	Pd1—Cl1^i^	2.3188 (6)
			
N2—C01—N1	104.54 (11)	C13—C14—C19	121.28 (12)
N2—C01—Pd1	128.86 (10)	C14—C15—C16	121.50 (14)
N1—C01—Pd1	126.59 (10)	C14—C15—H15	119.3
C03—C02—N1	106.89 (12)	C16—C15—H15	119.3
C03—C02—H02	126.6	C17—C16—C15	118.74 (13)
N1—C02—H02	126.6	C17—C16—C20	120.87 (13)
C02—C03—N2	106.60 (13)	C15—C16—C20	120.39 (13)
C02—C03—H03	126.7	C16—C17—C18	121.92 (13)
N2—C03—H03	126.7	C16—C17—H17	119
N1—C04—C05	113.17 (11)	C18—C17—H17	119
N1—C04—H04A	108.9	C17—C18—C13	117.17 (13)
C05—C04—H04A	108.9	C17—C18—C21	121.37 (12)
N1—C04—H04B	108.9	C13—C18—C21	121.42 (12)
C05—C04—H04B	108.9	C14—C19—H19A	109.5
H04A—C04—H04B	107.8	C14—C19—H19B	109.5
N3—C05—C04	114.26 (12)	H19A—C19—H19B	109.5
N3—C05—H05A	108.7	C14—C19—H19C	109.5
C04—C05—H05A	108.7	H19A—C19—H19C	109.5
N3—C05—H05B	108.7	H19B—C19—H19C	109.5
C04—C05—H05B	108.7	C16—C20—H20A	109.5
H05A—C05—H05B	107.6	C16—C20—H20B	109.5
O1—C06—N3	122.72 (14)	H20A—C20—H20B	109.5
O1—C06—C07	121.78 (13)	C16—C20—H20C	109.5
N3—C06—C07	115.50 (12)	H20A—C20—H20C	109.5
C08—C07—C12	119.67 (14)	H20B—C20—H20C	109.5
C08—C07—C06	118.15 (13)	C18—C21—H21A	109.5
C12—C07—C06	122.18 (14)	C18—C21—H21B	109.5
C09—C08—C07	120.36 (15)	H21A—C21—H21B	109.5
C09—C08—H08	119.8	C18—C21—H21C	109.5
C07—C08—H08	119.8	H21A—C21—H21C	109.5
C10—C09—C08	119.78 (16)	H21B—C21—H21C	109.5
C10—C09—H09	120.1	C01—N1—C02	110.93 (12)
C08—C09—H09	120.1	C01—N1—C04	125.88 (11)
C09—C10—C11	120.16 (15)	C02—N1—C04	123.18 (11)
C09—C10—H10	119.9	C01—N2—C03	111.05 (11)
C11—C10—H10	119.9	C01—N2—C13	125.47 (11)
C10—C11—C12	120.35 (15)	C03—N2—C13	123.48 (12)
C10—C11—H11	119.8	C06—N3—C05	122.04 (12)
C12—C11—H11	119.8	C06—N3—H1	117.2 (13)
C11—C12—C07	119.66 (15)	C05—N3—H1	117.1 (13)
C11—C12—H12	120.2	C01^i^—Pd1—C01	180
C07—C12—H12	120.2	C01^i^—Pd1—Cl1	92.45 (4)
C14—C13—C18	122.83 (12)	C01—Pd1—Cl1	87.55 (4)
C14—C13—N2	119.03 (12)	C01^i^—Pd1—Cl1^i^	87.55 (4)
C18—C13—N2	118.10 (12)	C01—Pd1—Cl1^i^	92.45 (4)
C15—C14—C13	117.61 (12)	Cl1—Pd1—Cl1^i^	180
C15—C14—C19	121.11 (13)		
			
N1—C02—C03—N2	−0.59 (16)	C14—C13—C18—C21	171.86 (12)
N1—C04—C05—N3	37.27 (16)	N2—C13—C18—C21	−5.84 (19)
O1—C06—C07—C08	−34.5 (2)	N2—C01—N1—C02	−0.38 (15)
N3—C06—C07—C08	144.81 (14)	Pd1—C01—N1—C02	−178.84 (10)
O1—C06—C07—C12	145.30 (15)	N2—C01—N1—C04	178.29 (12)
N3—C06—C07—C12	−35.44 (19)	Pd1—C01—N1—C04	−0.18 (19)
C12—C07—C08—C09	1.3 (2)	C03—C02—N1—C01	0.62 (16)
C06—C07—C08—C09	−178.98 (14)	C03—C02—N1—C04	−178.08 (13)
C07—C08—C09—C10	−1.3 (2)	C05—C04—N1—C01	51.09 (18)
C08—C09—C10—C11	0.1 (3)	C05—C04—N1—C02	−130.40 (14)
C09—C10—C11—C12	1.3 (2)	N1—C01—N2—C03	0.00 (15)
C10—C11—C12—C07	−1.3 (2)	Pd1—C01—N2—C03	178.42 (10)
C08—C07—C12—C11	0.1 (2)	N1—C01—N2—C13	179.11 (12)
C06—C07—C12—C11	−179.70 (13)	Pd1—C01—N2—C13	−2.47 (19)
C18—C13—C14—C15	3.7 (2)	C02—C03—N2—C01	0.38 (17)
N2—C13—C14—C15	−178.65 (12)	C02—C03—N2—C13	−178.75 (13)
C18—C13—C14—C19	−176.21 (13)	C14—C13—N2—C01	85.73 (17)
N2—C13—C14—C19	1.47 (19)	C18—C13—N2—C01	−96.48 (16)
C13—C14—C15—C16	0.6 (2)	C14—C13—N2—C03	−95.26 (16)
C19—C14—C15—C16	−179.50 (13)	C18—C13—N2—C03	82.53 (17)
C14—C15—C16—C17	−2.6 (2)	O1—C06—N3—C05	9.0 (2)
C14—C15—C16—C20	178.11 (13)	C07—C06—N3—C05	−170.29 (12)
C15—C16—C17—C18	0.4 (2)	C04—C05—N3—C06	57.45 (18)
C20—C16—C17—C18	179.69 (13)	N2—C01—Pd1—Cl1	−106.64 (12)
C16—C17—C18—C13	3.61 (19)	N1—C01—Pd1—Cl1	71.46 (11)
C16—C17—C18—C21	−173.97 (13)	N2—C01—Pd1—Cl1^i^	73.36 (12)
C14—C13—C18—C17	−5.72 (19)	N1—C01—Pd1—Cl1^i^	−108.54 (11)
N2—C13—C18—C17	176.58 (11)		

Symmetry code: (i) −*x*+2, −*y*, −*z*+2.

### Hydrogen-bond geometry (Å, º)

**Table:**

*D*—H···*A*	*D*—H	H···*A*	*D*···*A*	*D*—H···*A*
N3—H1···Cl1^i^	0.797 (19)	2.549 (19)	3.3181 (15)	162.6 (17)
C09—H09···O1^ii^	0.95	2.49	3.386 (2)	157
C10—H10···O1^iii^	0.95	2.44	3.201 (2)	137
C19—H19*C*···Cl1	0.98	2.81	3.772 (2)	167
C21—H21*B*···Cl1^i^	0.98	2.78	3.567 (2)	137

Symmetry codes: (i) −*x*+2, −*y*, −*z*+2; (ii) −*x*+1, *y*+1/2, −*z*+3/2; (iii) *x*, −*y*+1/2, *z*+1/2.

## References

[bb1] Altomare, A., Burla, M. C., Camalli, M., Cascarano, G. L., Giacovazzo, C., Guagliardi, A., Moliterni, A. G. G., Polidori, G. & Spagna, R. (1999). *J. Appl. Cryst.* **32**, 115–119.

[bb2] Arduengo, A. J. III, Harlow, R. H. & Kline, M. (1991). *J. Am. Chem. Soc.* **113**, 361–363.

[bb3] Bruker (2007). *APEX2*, *SAINT-Plus* and *SADABS* Bruker AXS Inc., Madison, Wisconsin, USA.

[bb4] Farrugia, L. J. (1999). *J. Appl. Cryst.* **32**, 837–838.

[bb5] Fu, C.-F., Lee, C.-C., Liu, Y.-H., Peng, S.-M., Warsink, S., Elsevier, C. J., Chen, J.-T. & Liu, S.-T. (2010). *Inorg. Chem.* **49**, 3011–3018. 10.1021/ic9025188 20143789

[bb6] Hahn, F. E. & Jahnke, M. C. (2008). *Angew. Chem. Int. Ed.* **47**, 3122–3172. 10.1002/anie.200703883 18398856

[bb7] Macrae, C. F., Bruno, I. J., Chisholm, J. A., Edgington, P. R., McCabe, P., Pidcock, E., Rodriguez-Monge, L., Taylor, R., van de Streek, J. & Wood, P. A. (2008). *J. Appl. Cryst.* **41**, 466–470.

[bb8] Meij, A. M. M., Otto, S. & Roodt, A. (2005). *Inorg. Chim. Acta*, **358**, 1005–1011.

[bb9] Sheldrick, G. M. (2008). *Acta Cryst.* A**64**, 112–122. 10.1107/S0108767307043930 18156677

[bb10] Wang, H. M. J. & Lin, I. J. B. (1998). *Organometallics*, **17**, 972–975.

[bb11] Warsink, S., Hauwert, P., Siegler, M. A., Spek, A. L. & Elsevier, C. J. (2009). *Appl. Organomet. Chem.* **23**, 225–228.

[bb12] Warsink, S., van Aubel, C. M. S., Weigand, J. J., Liu, S.-T. & Elsevier, C. J. (2010). *Eur. J. Inorg. Chem.* **35**, 5556–5562.

